# Efficacy and Feasibility of Short-Stretch Compression Therapy for Filarial Lymphedema in Sri Lanka

**DOI:** 10.4269/ajtmh.23-0496

**Published:** 2024-03-26

**Authors:** Jaimee M. Hall, Nirmitha Lalindi De Silva, Janaka Ruben, Sandani S. Thilakarathne, Thishan Channa Yahathugoda, Philip J. Budge

**Affiliations:** ^1^ Department of Pediatrics, Washington University in St. Louis, St. Louis, Missouri;; ^2^ Department of Parasitology, Faculty of Medicine, University of Ruhuna, Galle, Sri Lanka;; ^3^ Department of Medicine, Washington University in St. Louis, St. Louis, Missouri

## Abstract

The WHO-recommended essential package of care (EPC) for filarial limb lymphedema consists of daily limb washing, entry lesion management, limb protection, exercises, and elevation. Decongestive therapy (DT) with compression bandaging by trained lymphedema therapists adds additional benefit but is unavailable for most in low- and middle-income countries (LMICs). To determine whether DT using self-adjustable, short-stretch compression garments (SSCG), prefitted using portable, three-dimensional infrared imaging (3DII), would be effective and feasible in LMIC settings, we conducted a pilot 6-week, interventional, single-group, open-label pilot study in Galle, Sri Lanka. Ten participants with Dreyer stage 3 lymphedema used SSCG for 2 weeks after a 4-week lead-in EPC period. Effect of EPC and compression on quality of life was assessed using the 12-item WHO Disability Assessment Schedule 2.0 (WHODAS 2.0). Median participant age was 73 years (range: 32–85 years). Median percent limb volume reduction due to compression was 11.3% (range: 1.1–27.2%). WHODAS 2.0 scores did not change significantly between enrollment and study end. Garment acceptability was high throughout the study. These results provide proof of concept for 3DII-enabled SSCG in LMICs where trained therapists for filarial lymphedema may not be available.

## INTRODUCTION

Lymphatic filariasis (LF) is a tropical, mosquito-borne parasitic infection affecting more than 50 million people and causing chronic, disabling, disfiguring, and stigmatizing limb lymphedema in more than 1 million people globally.^1^^,^^2^ Disease progression is driven by recurrent secondary bacterial/fungal skin infections, termed acute dermatolymphangioadenitis (ADLA). Routine management for filarial lymphedema in low- and middle-income countries (LMICs) consists of a WHO-recommended essential package of care (EPC) of daily washing, skin care, elevation, exercises, and proper footwear, which together prevent ADLA episodes and slow lymphedema advancement.^3^^–^^6^ In high-income countries, limb compression managed by trained lymphedema therapists, is a standard component of lymphedema management that reduces limb volume, disability, and disfigurement and improves quality of life (QOL).^7^^–^^9^ Typical compression therapy consists of two phases: 1) initial volume reduction accomplished through comprehensive decongestive therapy (DT), which includes daily therapist-directed compression bandaging, followed by 2) maintenance accomplished through static compression once a volume reduction plateau has been reached.

Unfortunately, in most low-resource settings, DT is not feasible because compression garments and/or trained therapists are not readily available. Prior studies for filarial lymphedema in LMIC settings found that compression improved limb volumes but may increase ADLA episodes in the absence of concomitant adherence to EPC practices.^3^^,^^10^

The recent availability of customizable, short stretch compression garments (SSCG), which allow for self-adjustment by patients, prompted us to consider whether these garments could provide an acceptable substitute for DT in Sri Lanka. We have previously validated a novel, compact, portable, 3D infrared imaging (3DII) tool to measure limb volume changes over time in filarial lymphedema.^11^^,^^12^ The virtual 3D point cloud models generated by 3DII can be used by commercial compression garment manufacturers to custom fit garments for compression induction and maintenance. In this study, we sought to determine whether patient-directed use of SSCG, prefitted using 3DII, would be an effective and acceptable adjunct to the EPC for filarial lymphedema in a tropical setting.

## MATERIALS AND METHODS

### Population and setting.

This study was conducted at the Filariasis Research Training and Services Unit (FRTSU), Department of Parasitology, Faculty of Medicine, University of Ruhuna in Galle, Sri Lanka. Participant visits took place at the FRTSU clinic and in subject homes. FRTSU is a government funded LF clinic that provides care for more than 300 lymphedema patients in southwestern Sri Lanka. FRTSU patients with Dreyer stage 3 lymphedema (characterized by increased limb volume accompanied by persistent skin creases, or “shallow folds” that do not resolve with overnight elevation)^13^ were invited to participate. Additional inclusion criteria included age ≥18 years, willingness and ability to adhere to EPC measures, and the ability to stand for 2 minutes unassisted. Exclusion criteria included open lower extremity wounds, pregnancy, or any use of compression garments in the preceding 2 months.

### Study design, materials, and procedures.

**Figure 1.**
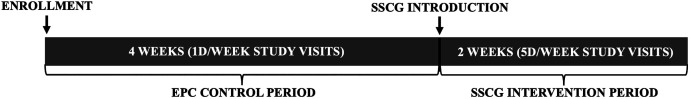
Study timeline. The study consisted of an essential package of care (EPC) control period for 4 weeks, in which participants were visited weekly by the study team. This EPC control period was followed by a short stretch compression garment (SSCG) intervention period for 2 weeks, in which the participants were visited 5 days a week by the study team.

This 6-week, interventional, single group, open-label, pilot study, was conducted from January–March 2023, and included a 4-week lead-in period of EPC, followed by a 2-week intervention period of daily SSCG use (Figure 1). After providing informed consent, participants were trained to perform the EPC, including daily washing, drying, management of entry lesions, limb elevation, exercise, and shoe use outside/inside the home to protect limbs using the WHO’s “New Hope for People with Lymphedema” manual.^14^ Hygiene supplies, including a wash basin, soap, and towel, were provided at initial study visit. Complete study schedule of events is shown in Supplemental Appendix A. During the EPC control period, the study team visited each participant in their home weekly. This lead-in period allowed participants to demonstrate good EPC practices and provided multiple pre-compression volume measurements to establish a robust mean baseline volume.

### Intervention.

Following 4 weeks of EPC, compression therapy was introduced using the Circaid^®^ Reduction Kit™ SSCGs and adjustable Circaid^®^ Comfort PAC Band (donated by medi USA, Whitsett, NC). These compression products are adjustable, inelastic, (short-stretch) garments composed of Breathe-O-Prene^®^, a material that allows for garment breathability. Adjustable, overlapping Velcro straps with built-in tension markers allow for control and modification of therapeutic tension level. Photos of the garment products can be seen in Supplemental Figure A. The garments were sized using 3DII point cloud models and measurements captured using the LymphaTech™ handheld mobile device (LymphaTech, Atlanta, GA) and fitted by trained study staff at the first compression visit. At this visit and subsequent visits, patients were instructed on garment donning and tension adjustments and performed these adjustments themselves with guidance. Patients were instructed to wear the garment at all times, including overnight, to provide maximum benefit, aside from 1 hour per day to perform hygiene. During the intervention period, home visits occurred 5 days a week. Garment instruction was repeated at follow-up visits which allowed for gradual escalation in participant confidence and independence with self-garment use. The study team followed a similar daily travel route allowing for participants to anticipate similar arrival times to time garment removal and perform daily hygiene. After study conclusion, participants were encouraged to continue SSCG use until custom static garments were made available to them by the study team.

### Limb volume measurements.

We used the 3DII (LymphaTech^®^) scanner to obtain limb measurements as previously described.^11^^,^^12^ Briefly, the patient stands stationary within a flat, open space with feet approximately shoulder-width apart with the examiner standing anterior to them ∼2 to 3 feet away. The examiner positions the tablet so that the patient’s legs from above the knee to the floor, are positioned within a sizing box visible on the screen. The examiner initiates data capture, then walks in a complete counterclockwise circle around the standing patient, allowing the device to capture infrared rays refracted from the surface of the patient’s legs, creating a 3D point cloud model. Once the examiner has captured the complete surface area (usually within 30 to 60 seconds), data capture is stopped and the examiner visually inspects the 3D patient limb reconstruction to ensure an accurate reflection of the limb is depicted. The examiner then saves the model, which then prompts the LymphaTech application to calculate total limb volume and circumference measurements from the 3D model. In this study, leg volume was calculated from a height of 35 cm to the floor. Participants were scanned three times at each visit and reported volume and circumference measurements represent the average of these three measurements.

### Disability and acceptability surveys.

Participants’ perceived disability was measured via the WHO Disability Assessment Schedule 2.0 (WHODAS 2.0) 12-item, interviewer administered version (Supplemental Appendix B).^15^ This validated instrument developed by the WHO evaluates six life domains that contribute to QOL such as cognition, mobility, self-care, getting along, life activities, and participation.^16^ Each item is presented in question form such as “In the past 30 days, how much difficulty have you had in: Standing for long periods such as 30 minutes?” and responses measured on a 5-point Likert-type scale, ranging from 1 (“none”) to 5 (“extreme or cannot do”). A total score out of a 60-point maximum is calculated, with higher scores equating to higher perceived disability. This survey was used at enrollment (day –28), precompression (day 0), and postcompression (day 14) by the same study team member.

We assessed garment acceptability using an acceptability survey created for this study (Supplementary Appendix C). The survey’s four questions focused on *anticipated* garment benefit and feasibility precompression and *perceived* garment benefit and feasibility at 1 week and again at 2 weeks into compression. Survey responses were collected by a trained interviewer, who read each statement, such as “I am willing to wear the compression garment whenever I am not washing my leg,” and then asked for the level of participant agreement with the statement using a 5-point Likert scale ranging from 1 (“strongly disagree”) to 5 (“strongly agree”). Each score is calculated out of a 20-point maximum. Higher scores equate to higher acceptability and lower scores to lower acceptability. This survey was used precompression (day 0), 1 week into compression (day 7), and 2 weeks into compression (day 14) by the same study team member.

### Patient interviews and diaries.

Informal, qualitative short interviews were conducted with participants at each study visit during the compression intervention period (days 1–14) to evaluate participant garment experiences and responses immediately transcribed onto case report forms. WHO-recommended EPC and SSCG adherence were recorded by participants in home diaries, reviewed by study team members at each study visit, and data recorded onto case report forms (Supplemental Appendix D).

## STATISTICAL ANALYSES

Extracted data were first collected in the field on paper case report forms and data subsequently secured in a locked storage cabinet at the University of Ruhuna. Data was then manually entered into a secure, web-based, password-protected, Health Insurance Portability and Accountability Act–compliant REDCap^®^ electronic data capture tool.^17^^,^^18^ Statistical analyses were performed in IBM SPSS Statistics For Windows, Version 28 (IBM Corp., Armonk, NY).^19^ Last value carried forward method was used for missing data, which occurred for one participant at one follow-up study visit and was used for both leg volume and circumference. For descriptive analyses, categorical variables are reported as proportions and continuous variables as means with standard deviation or medians with range. Within-subject repeated measures of change in leg volume, leg circumference, and QOL scores were assessed using the Friedman’s test. Correlations were assessed using Spearman’s correlation testing. Significance was defined using a two-tailed alpha of 0.05.

Percent reduction in limb size was defined as (baseline mean – postcompression mean) / baseline mean. Baseline mean was defined as the average of precompression measurements (days –28, –21, –14, –7, day 0). Postcompression mean was defined as the average leg volumes or circumferences during the last week of compression (days 8–14).

## RESULTS

### Demographics and history.

Eleven participants, including six men and five women, were enrolled, and one male participant was lost to attrition. Median age among final participants was 73 years (range: 32–85); selected demographics and medical history are shown in Table 1. Most participants had lymphedema of more than 5 years’ duration, and three participants (30%) had experienced one ADLA episode in the 2 months preceding the study. All reported complete adherence to once-daily limb washing in the month preceding the start of the study.

**Table 1:** Demographics and medical history, *N* = 10

Demographics/History	*n* (%)
Age (years)	
30–49	2 (20)
50–69	2 (20)
70+	6 (60)
Median age (range)	73 (32–85)
Sex	
Males	5 (50)
Females	5 (50)
Occupation	
Housewife	4 (40)
self-employed*	2 (20)
Retired security officer	2 (20)
Retired government clerical officer	1 (10)
Painter	1 (10)
Medical comorbidities	
Hypertension	2 (20)
Diabetes mellitus	2 (20)
Hyperlipidemia	2 (20)
Mastectomy	1 (10)
Ischemic heart disease	1 (10)
Osteoarthritis	1 (10)
Hydrocele	1 (10)
Lymphedema duration (years)	
<5	2 (20)
5–19	4 (40)
≥20	4 (40)
ADLA episodes in 2 months preceding study	
0	7 (70)
1	3 (30)
Hygiene adherence during month preceding study (no missed days)	10 (100)

ADLA = acute dermatolymphangioadenitis.

* Seamstress, coconut oil manufacturer, and salesman.

### Lower extremity volume changes.

**Figure 2.**
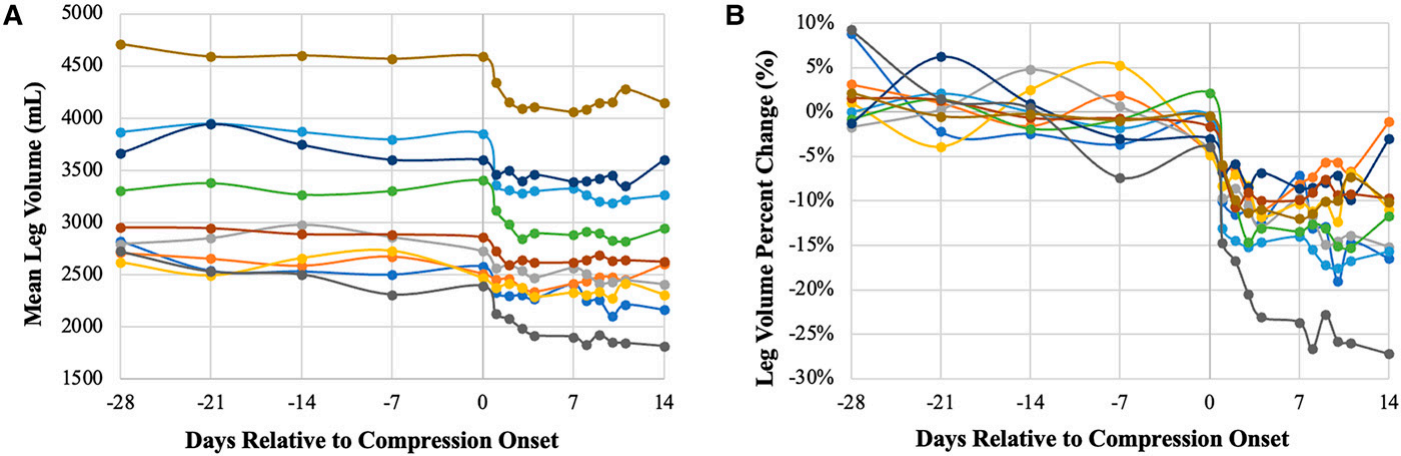
Lower extremity volume change over time represented in mean volume and normalized percent change. (**A**) Within-subject mean lower extremity volume change over time relative to days to compression (day 0). Each participant is represented by a distinct line color. (**B**) Within-subject mean lower extremity volume percent change normalized to precompression average volume change over time relative to days to compression (day 0). Each participant is represented by a distinct line color.

**Figure 3.**
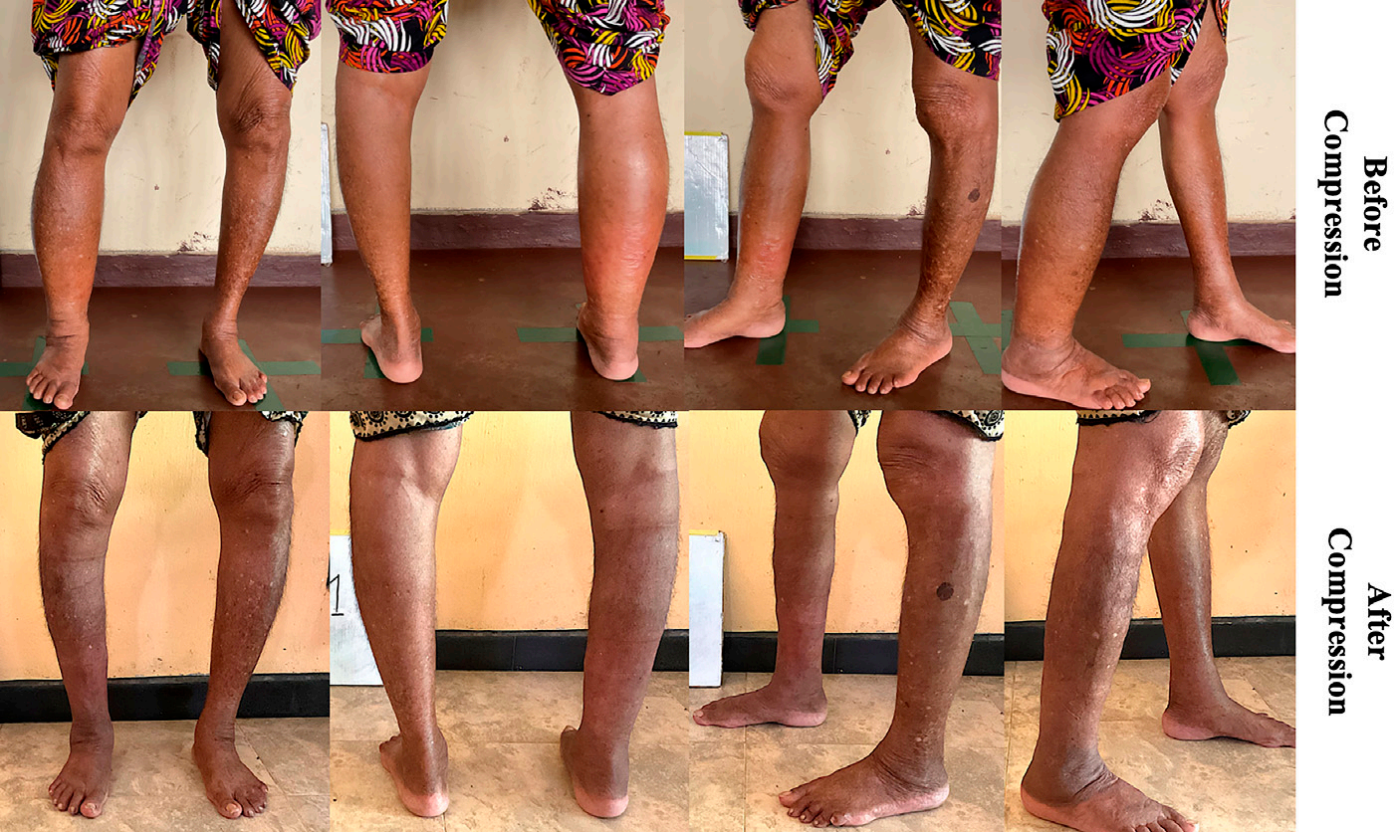
Clinical photographs comparing affected leg appearance enrollment to post-compression. Clinical photographs for an individual subject with an affected right lower extremity, comparing enrollment (day −28, top row) to post-compression (day 14, bottom row) appearance from anterior, posterior, left lateral, and right lateral views.

Median precompression leg volume among all participants was 2,874 mL (range: 2,493–4,614). There was a slight downward trend in limb volume during the lead-in period, followed by a significant reduction in volume during compression (Figure 2). Postcompression mean volume reduction was –397 mL or –12.7% (range: –640 to –139 mL or –26.7 to –5.3%). The maximal mean percent volume reduction occurred on day 10 (Supplemental Figure B). Median time to volume reduction plateau was 7.5 days (range: 2–10) (Supplemental Table B). No significant relationship between lymphedema duration and percent volume change was seen (*r* = –0.252; 95% CI: –0.770 to 0.467; *P* = 0.483) (Supplemental Figure C). Figure 3 illustrates limb size reduction from enrollment (day –28) to postcompression (day 14) for one participant.

### Disability score changes.

Median enrollment WHODAS 2.0 score was 13.5 (range: 12–36) and postcompression was 14 (range: 12–32) and no significant differences of within-subject repeated measures was seen (*P* = 0.587) (Table 2).

**Table 2:** WHODAS 2.0 short form disability scores* at landmark visits

Subject	Enrollment (day −28)	Precompression (day 0)	Postcompression (day 14)	Overall Score Change (day −28 to 14)
A	29	29	30	1
B	28	33	20	−8
C	13	12	12	−1
D	12	12	12	0
E	14	14	15	1
F	12	13	12	0
G	12	13	13	1
H	12	13	12	0
I	36	38	32	−4
J	17	16	18	1
Median (range)	13.5 (12–36)	13.5 (12–38)	14 (12–32)	0 (–8 to 1)

* A total score is calculated out of a maximum of 60 points, with higher scores equating to a higher level of disability. The disability survey was used at enrollment (day −28), precompression (day 0), and postcompression (day 14). Overall score change was calculated between enrollment (day −28) and postcompression (day 14). A positive change indicates a higher perceived level of disability and a negative change a lower perceived level of disability.

### Hygiene and compression adherence.

There were no reported missed days of hygiene during the 6-week study. One of 10 participants averaged ≥23 hours per day of SSCG, whereas all participants averaged >21 hours per day of use. Overall median SSCG use was 22.75 hours (range: 21.5–23.0) (Supplemental Table B).

### Garment acceptability.

Median acceptability score precompression was 18 points (range: 16–20), 1 week into compression was 20 (range: 17–20), and postcompression was 18.5 (range: 14–20) (Table 3). Participant garment experiences from interviews are summarized in Table 4.

**Table 3:** Acceptability scores* precompression, during compression, and postcompression

Subject	Precompression (day 0)	One Week of Compression (day 7)	Postcompression (day 14)
A	18	20	16
B	18	20	19
C	16	20	16
D	19	20	20
E	17	18	19
F	17	20	19
G	20	20	18
H	18	18	18
I	19	20	20
J	17	17	14
Median (range)	18 (16–20)	20 (17–20)	18.5 (14–20)

* Acceptability scores were measured via a de novo acceptability survey used precompression (day 0), 1 week into compression (day 7), and 2 weeks into compression (day 14). Precompression (day 0) statements focused on anticipated garment benefit and feasibility, whereas during compression (days 7 and 14) statements focused on perceived benefit and feasibility. The surveys at 1 and 2 weeks into compression were the same. Higher scores equate to a higher level of acceptability and lower scores to a lower level of acceptability. Scores were out of a maximum of 20 points.

**Table 4:** Qualitative garment comments

Type of Comment	Comfort/Support	Ease/Willingness	Stigma/Use Outside Home
Positive	“Not sweaty or itchy” “No sleep disturbance or discomfort” “Feels supportive when walking” “Leg feels lighter” “Provides a sense of relief” “Notable improvement in leg”	“Confident to self-don/doff” “Confident to don with assistance” “Does not disrupt day to day life”	“Not worried about stigma, more focused on lymphedema improvement” “Okay with being asked about it outside home by strangers” “Rode bike into town with it on” “Hiked with it on” “Wore to a wedding, well covered by clothing” “Wore to work regularly”
Negative	“Mild pressure, mild discomfort, mild itchiness” “Bulky with sleep” “Mildly restricts ankle movement for recommended exercises” “All day use uncomfortable” “Difficult/uncomfortable to self-don/doff, adjust straps”	“Foot piece difficult to fully cover with clothing” “Difficult to wear when handwashing clothes/at risk of getting wet” “Wish was darker in color to match skin tone”	“Hesitant to leave house” “Took foot piece off when outside to avoid soiling foot piece” “Can be annoying when strangers ask about its purpose outside home” “Fear of wearing outside home due to stigma”

### Leg circumference changes.

**Figure 4.**
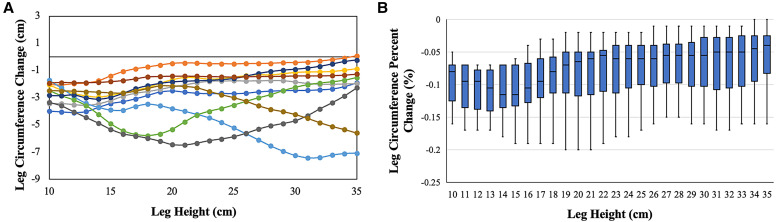
Lower extremity circumference change at varying leg heights precompression to postcompression: (**A**) mean differences and (**B**) percent change. Within-subject circumference differences are calculated by subtracting average postcompression circumference (days 8–14) from average precompression circumference (day –28 to day 0). In panel A, each participant is represented by a distinct line color.

To determine whether the compressive effect of SSCG use differed by anatomic location, we examined leg circumferences derived from the 3DII models. Circumference reduction pattern varied by individual, but mean circumference reduction was greatest at a height of 13 to 16 cm, corresponding to just above the ankle (Figure 4). This is in keeping with our selection of stage 3 patients, because the defining characteristic of this lymphedema stage is the presence of shallow skin folds, the majority of which occur at the ankle.

## DISCUSSION

Morbidity management for filarial limb lymphedema remains an ongoing global challenge. Most affected individuals do not have reasonable access to resources needed for DT, including lymphedema therapists, inelastic bandages or durable compression garments, and cost- and time-efficient lymphedema measurement tools.^2^ In this small pilot study, we sought to determine whether SSCG, prefitted using a low-cost, portable, lymphedema measurement tool (3DII), would be acceptable and effective in reducing limb volume in patients with filarial lymphedema in Sri Lanka. We found that 2 weeks of SSCG compression therapy significantly reduced limb volume and circumference and was acceptable to participants, as evidenced by acceptability survey scores, adherence to therapy, absence of ADLA episodes, and no increase in WHODAS 2.0 scores.

Most participants reported the garment as consistently comfortable, supportive, easy to use, and beneficial for their lymphedema. We observed that participants were motivated to continue use when provided estimated volume changes at serial visits. Mild discomfort associated with garment pressure or pruritus was rare and easily augmented with tension reduction or emollient application after garment removal, respectively. Only a few participants found use during sleep or all day to be somewhat cumbersome. At least one patient felt a sense of stigma in wearing the garment outside the home, whereas another was pleased to wear the garment in public because it provided visible evidence of improved care for his lymphedema.

Our study is subject to several limitations. First, because this was a small pilot feasibility study, our sample size was small and included only participants with stage 3 lymphedema. Further studies will be required to determine whether patients with more advanced lymphedema would derive a similar clinical benefit and achieve a similar acceptance level as that seen in our cohort. Second, this study was too short in duration to provide meaningful data on ADLA episodes. Increased ADLA rates with compression garment use were observed in a prior filarial lymphedema study, potentially due to an initial lack of adherence to optimal hygiene measures.^3^ In these prior studies, it is unclear what housing structures these patients had, which could contribute to ability to keep compression garments and limbs clean. Although the absence of ADLA episodes in our study is reassuring, a longer follow-up with more participants would be essential to determine true SSCG effects on ADLA episode rate. Third, adherence to garment use was not directly monitored. The reported average garment use of more than 21 hours per day in our study is longer than that seen in prior filarial lymphedema compression studies, which reported 0 to 10 hours during the day and 1 to 10 hours overnight.^10^ Self-reported adherence is subject to recall and desirability bias. Thus, we cannot confirm that those who benefited less than anticipated actually wore the garments as reported. A few participants in our study experienced only modest response, and some had notable volume increases over the final 3 study days (a holiday weekend). Whether this variability and end-of-study increase may be due to unreported decreased garment use is unclear. Another possibility is that unmeasured physiologic differences such as extent of tissue fibrosis or residual lymphatic function^20^^,^^21^ are responsible for the range of compression responses. Future studies, inclusive of higher lymphedema stages, could consider incorporation of adjunctive lymphedema measurement techniques such as bioimpedance to advance and customize compression implementation.^22^^,^^23^ Finally, most of our participants were retired or worked from their homes and lived in multigenerational households, which likely facilitated SSCG adherence and ability to keep garments clean given their houses consisted of reliable flooring. It is unclear whether the same results could have been achieved in those working outside of the home or in older individuals not living within multigenerational homes.

This study was made possible by donated resources. The hardware required for the portable 3DII used in our study (tablet, infrared sensor, and proprietary software) in the U.S. market would cost between $1,000 and $2,000. Likewise, the retail cost of the SSCG kits used in our study range from $100 to $200. We acknowledge these resources are currently no more accessible to most patients suffering from filarial lymphedema than is therapist-directed bandaging. However, cost could potentially be lowered via donation or local manufacturing, and SSCG may prove a more practical and cost-effective way of providing decongestive therapy than training and supplying new lymphedema therapists where none currently exist. 3DII and SSCG are technologies that are easy to use with relatively minimal initial training; thus, their use should be well within the capacity of the staff of national lymphedema management programs and even community volunteers. Furthermore, SSCG are self-adjustable by patients after initial instruction and only require skilled staff to resize the base garment in response to limb reduction until a volume reduction plateau is reached. Should further studies validate our preliminary results and show 3DII-fitted SSCG therapy is beneficial for individuals severely affected by filarial lymphedema, it may prove a valuable option for lymphedema management in LMIC if resources can be found to make it available.

Despite its limitations, this study has important implications. This is the first study to demonstrate the feasibility of 3DII-enabled SSCG in lieu of therapist-directed bandaging for filarial lymphedema in a LMIC setting. Although this study included only a small number of participants and all with stage 3 lymphedema, it is clear that self-adjustable, 3DII-enabled SSCG can be implemented in a tropical setting. This suggests that patient-adjusted SSCG, if made available, could provide the benefits of DT to affected lymphedema patients living where there is no access to trained lymphedema therapists. It is probable that patients with larger limb volumes will derive more benefit from compression than those in our study. Further studies of longer duration, greater sample size, with diverse limb stages, and inclusive of diverse home settings, will be required to test this hypothesis.

## Supplemental Materials

10.4269/ajtmh.23-0496 Supplemental Materials
