# Supplementary material for: Efficacy and Feasibility of Short-Stretch Compression Therapy for Filarial Lymphedema in Sri Lanka
Source: Am J Trop Med Hyg. 2024 Mar 26;110(5):936–42. doi: 10.4269/ajtmh.23-0496 (PMC11066340; doi:10.4269/ajtmh.23-0496)
Supplement: Supplemental Materials [file tpmd230496.SD1.pdf]

Appendix A: Study Schedule

**Schedule of Activities:**					
	**Visit 1:** Enrollment	**Visits 2-4:** Pre-Compression	**Visit 5:** Garment Fitting	**Visit 6-14:** Post-Compression	**Visit 15:** End of Study
**Days of Study:**	1±2	8±2, 15±2, 22±2	29±5	Post-Compression Days 2-13*	Post-Compression Days 14±2
**Informed Consent**	X				
**In-/Exclusion Criteria**	X				
**Demographic Data**	X				
**Medical History (Hx)**	X				
**Focused Physical Examination**	X	X	X	X	X
**Lymphedema (LE) Staging**	X				X
**Clinical Photographs**	X				X
**LE Hygiene Hx**	X	X	X	X	X
**ADLA Hx**	X	X	X	X	X
**LE Volume - LymphaTech®**	X	X	X	X	X
**WHO-DAS II Assessment**	X		X		X
**Acceptability Survey**			X	X (weekly)	X
**LE Hygiene Training/Review**	X	X	X	X (weekly)	X
**Hygiene Supplies Dispersed**	X				
**Compression Garment Fitting**			X		
**Compression Garment Adjustment**				X	X
**Compression Garment Hx**				X	X

*Larger range of days during this aggressive compression period to allow for frequent (5 days/week) serial visits but to account for lack of study visits expected to occur on weekend days.

WHODAS 2.0

WORLD HEALTH ORGANIZATION
DISABILITY ASSESSMENT SCHEDULE 2.0

12-item version, interviewer-administered

Introduction

This instrument was developed by the WHO *Classification, Terminology and Standards* team, within the framework of the WHO/National Institutes of Health (NIH) Joint Project on Assessment and Classification of Disability.

Before using this instrument, interviewers must be trained using the manual *Measuring Health and Disability: Manual for WHO Disability Assessment Schedule – WHODAS 2.0* (WHO, 2010), which includes an interview guide and other training material.

The versions of the interview available are as follows:

- 36-item – Interviewer-administered[a]
- 36-item – Self-administered
- 36-item – Proxy-administered[b]
- 12-item – Interviewer-administered[c]
- 12-item – Self-administered
- 12-item – Proxy-administered
- 12+24-item – Interviewer-administered

[a] A computerized version of the interview (*iShell*) is available for computer-assisted interviews or for data entry

[b] Relatives, friends or caretakers

[c] The 12-item version explains 81% of the variance of the more detailed 36-item version

For more details of the versions please refer to the WHODAS 2.0 manual *Measuring Health and Disability: Manual for WHO Disability Assessment Schedule – WHODAS 2.0* (WHO, 2010).

Permission to translate this instrument into any language should be obtained from WHO, and all translations should be prepared according to the WHO translation guidelines, as detailed in the accompanying manual.

For additional information, please visit www.who.int/whodas or contact:

Dr T Bedirhan Üstün
Classification, Terminology and Standards
Health Statistics and Informatics
World Health Organization (WHO)
1211 Geneva 27
Switzerland

Tel: + 41 22 791 3609
E-mail:ustunb@who.int

This questionnaire contains the interviewer-administered, 12-item version of WHODAS 2.0.

Instructions to the interviewer are written in bold and italics – do not read these aloud

Text for the respondent to hear is written in

standard print in blue.

Read this text aloud

Section 1 Face sheet

Complete items F1–F5 before starting each interview				
F1	Respondent identity number			
F2	Interviewer identity number			
F3	Assessment time point (1, 2, etc)			
F4	Interview date	_____ day	_____ month	_____ year
F5	Living situation at time of interview (circle only one)	Independent in community		1
		Assisted living		2
		Hospitalized		3

Please continue to next page ...

Section 2 Demographic and background information

This interview has been developed by the World Health Organization (WHO) to better understand the difficulties people may have due to their health conditions. The information that you provide in this interview is confidential and will be used only for research. The interview will take 5–10 minutes to complete.

For respondents from the general population (not the clinical population) say:

Even if you are healthy and have no difficulties, I need to ask all of the questions so that the survey is complete.

I will start with some background questions.

A1	***Record sex as observed***	Female	1
		Male	2
A2	How old are you now?	______ years	
A3	How many years in all did you spend studying in school, college or university?	______ years	
A4	What is your current marital status? ***(Select the single best option)***	Never married	1
		Currently married	2
		Separated	3
		Divorced	4
		Widowed	5
		Cohabiting	6
A5	Which describes your main work status best? ***(Select the single best option)***	Paid work	1
		Self-employed, such as own your business or farming	2
		Non-paid work, such as volunteer or charity	3
		Student	4
		Keeping house/ homemaker	5
		Retired	6
		Unemployed (health reasons)	7
		Unemployed (other reasons)	8
		Other (specify)______________ ____________________	9

Please continue to next page...

Section 3 Preamble

Say to respondent:

The interview is about difficulties people have because of health conditions.

Hand flashcard #1 to respondent

By health condition I mean diseases or illnesses, or other health problems that may be short or long lasting; injuries; mental or emotional problems; and problems with alcohol or drugs.

Remember to keep all of your health problems in mind as you answer the questions. When I ask you about difficulties in doing an activity think about...

Point to flashcard #1

- Increased effort
- Discomfort or pain
- Slowness
- Changes in the way you do the activity.

When answering, I'd like you to think back over the past 30 days. I would also like you to answer these questions thinking about how much difficulty you have had, on average, over the past 30 days, while doing the activity as you usually do it.

Hand flashcard #2 to respondent

Use this scale when responding.

Read scale aloud:

None, mild, moderate, severe, extreme or cannot do.

Ensure that the respondent can easily see flashcards #1 and #2 throughout the interview

Please continue to next page...

Section 4 Core questions

Show flashcard #2

In the past 30 days, how much difficulty did you have in:		None	Mild	Moderate	Severe	Extreme or cannot do
S1	Standing for long periods such as 30 minutes?	1	2	3	4	5
S2	Taking care of your household responsibilities?	1	2	3	4	5
S3	Learning a new task, for example, learning how to get to a new place?	1	2	3	4	5
S4	How much of a problem did you have joining in community activities (for example, festivities, religious or other activities) in the same way as anyone else can?	1	2	3	4	5
S5	How much have you been emotionally affected by your health problems?	1	2	3	4	5

In the past 30 days, how much difficulty did you have in:		None	Mild	Moderate	Severe	Extreme or cannot do
S6	Concentrating on doing something for ten minutes?	1	2	3	4	5
S7	Walking a long distance such as a kilometre [or equivalent]?	1	2	3	4	5
S8	Washing your whole body?	1	2	3	4	5
S9	Getting dressed?	1	2	3	4	5
S10	Dealing with people you do not know?	1	2	3	4	5
S11	Maintaining a friendship?	1	2	3	4	5
S12	Your day-to-day work/school?	1	2	3	4	5

H1	Overall, in the past 30 days, how many days were these difficulties present?	***Record number of days*** ____
H2	In the past 30 days, for how many days were you totally unable to carry out your usual activities or work because of any health condition?	***Record number of days*** ____
H3	In the past 30 days, not counting the days that you were totally unable, for how many days did you cut back or reduce your usual activities or work because of any health condition?	***Record number of days*** ____

This concludes our interview. Thank you for participating.

Health conditions:

- Diseases, illnesses or other health problems
- Injuries
- Mental or emotional problems
- Problems with alcohol
- Problems with drugs

Having difficulty with an activity means:

- Increased effort
- Discomfort or pain
- Slowness
- Changes in the way you do the activity

Think about the past 30 days only.

1	2	3	4	5
**None**	**Mild**	**Moderate**	**Severe**	**Extreme or cannot do**

Acceptability Survey

Barcode

Date: ____ / _____ / ______
DD MM YYYY

PRE-COMPRESSION (Visit 5)

Please read this line to the subject prior to helping them fill out the survey: <u>"Now, I would like you to tell me how much you disagree or agree with each of the following statements. There are no right or wrong answers. Please answer honestly."</u>

	Strongly Disagree	Disagree	Neither Agree or Disagree	Agree	Strongly Agree
I am willing to wear the compression garment whenever I am not washing my leg.	1	2	3	4	5
I am motivated to wear the compression garment.	1	2	3	4	5
The compression garment will reduce the size of my leg.	1	2	3	4	5
The compression garment will be easy to wear.	1	2	3	4	5

If any of the above answers are "strongly disagree" or "disagree" please provide a simple explanation as to why: ____

____

____

____

Name of person completing this form: ______________________________

Signature: ______________________ Date: ____ / ______ / ______
DD MM YYYY

Acceptability Survey

Barcode

Date: ____ / _____ / ______
DD MM YYYY

ONE WEEK INTO COMPRESSION (Visit 8)

Please read this line to the subject prior to helping them fill out the survey: "Now, I would like you to tell me how much you disagree or agree with each of the following statements. There are no right or wrong answers. Please answer honestly."

	Strongly Disagree	Disagree	Neither Agree or Disagree	Agree	Strongly Agree
I wore the compression garment whenever I was not washing my leg.	1	2	3	4	5
The compression garment is easy to use.	1	2	3	4	5
The compression garment is helping reduce the size of my leg.	1	2	3	4	5
I am satisfied with using the compression garment.	1	2	3	4	5

If any of the above answers are "strongly disagree" or "disagree" please provide a simple explanation as to why: ______________________________

____

____

____

Name of person completing this form: ______________________________

Signature: ______________________________ Date: ____ / ______ / ______
DD MM YYYY

Acceptability Survey

Barcode

Date: ____ / _____ / ______
DD MM YYYY

TWO WEEKS INTO COMPRESSION (Visit 14)

Please read this line to the subject prior to helping them fill out the survey: "Now, I would like you to tell me how much you disagree or agree with each of the following statements. There are no right or wrong answers. Please answer honestly."

	Strongly Disagree	Disagree	Neither Agree or Disagree	Agree	Strongly Agree
I wore the compression garment whenever I was not washing my leg.	1	2	3	4	5
The compression garment is easy to use.	1	2	3	4	5
The compression garment is helping reduce the size of my leg.	1	2	3	4	5
I am satisfied with using the compression garment.	1	2	3	4	5

If any of the above answers are "strongly disagree" or "disagree" please provide a simple explanation as to why: ____

____

____

____

Name of person completing this form: ____

Signature: ______________________________ Date: ____ / ______ / ______
DD MM YYYY

Supplemental Tables

A. Mean Affected Lower Extremity Volumes[a] & Volume Change[b] at Landmark Visits

Subject	Pre-Compression Mean (SD) Volume (mL) (Day -28 to Day 0)	Post-Compression Mean (SD) Volume (mL) (Day 8 to 14)	Overall Volume Change (mL)
**A**	2593 (131)	2198 (104)	-395
**B**	2628 (80)	2489 (65)	-139
**C**	2841 (94)	2442 (62)	-399
**D**	2595 (111)	2329 (49)	-266
**E**	3869 (54)	3229 (51)	-640
**F**	3333 (57)	2882 (46)	-451
**G**	3715 (143)	3445 (88)	-270
**H**	2907 (41)	2647 (24)	-260
**I**	2493 (157)	1853 (43)	-640
**J**	4614 (57)	4164 (75)	-450
**Median (range)**	**2874 (2493 to 4614)**	**2568 (1853 to 4164)**	**-397 (range -139 to -640)**

a. Pre-compression mean volume is calculated for each subject using the leg volume means at the five first visits pre-compression (day -28 to day 0). Post-compression mean volume is calculated for each subject using the leg volume means at the five last visits during compression (day 8 to day 14).
b. Overall volume change is calculated for each subject by subtracting post-compression mean volume from pre-compression mean volume. A positive value indicates a volume gain and a negative value a reduction.

B. Compression Garment Use[a] & Time to Volume Reduction Plateau[b]

Subject	Median Hours of Garment Use	Time to Volume Reduction Plateau (Days)
**A**	22.75	9
**B**	21.5	3
**C**	23.25	8
**D**	23	4
**E**	22.75	8
**F**	22.5	9
**G**	22.75	10
**H**	22.25	2
**I**	23	7
**J**	21.83	7
**Median (range)**	**22.75 (21.5-23)**	**7.5 (2-10)**

a. Median hours of compression garment use were calculated for each subject by calculating the median daily compression garment use in hours during the intervention period (day 0 to day 14).
b. Time to volume reduction plateau was calculated for each subject by measuring days from compression introduction (day 0) to the first day with mean lower extremity volume equal to or below the mean of the three lowest leg volumes.

Supplemental Figures

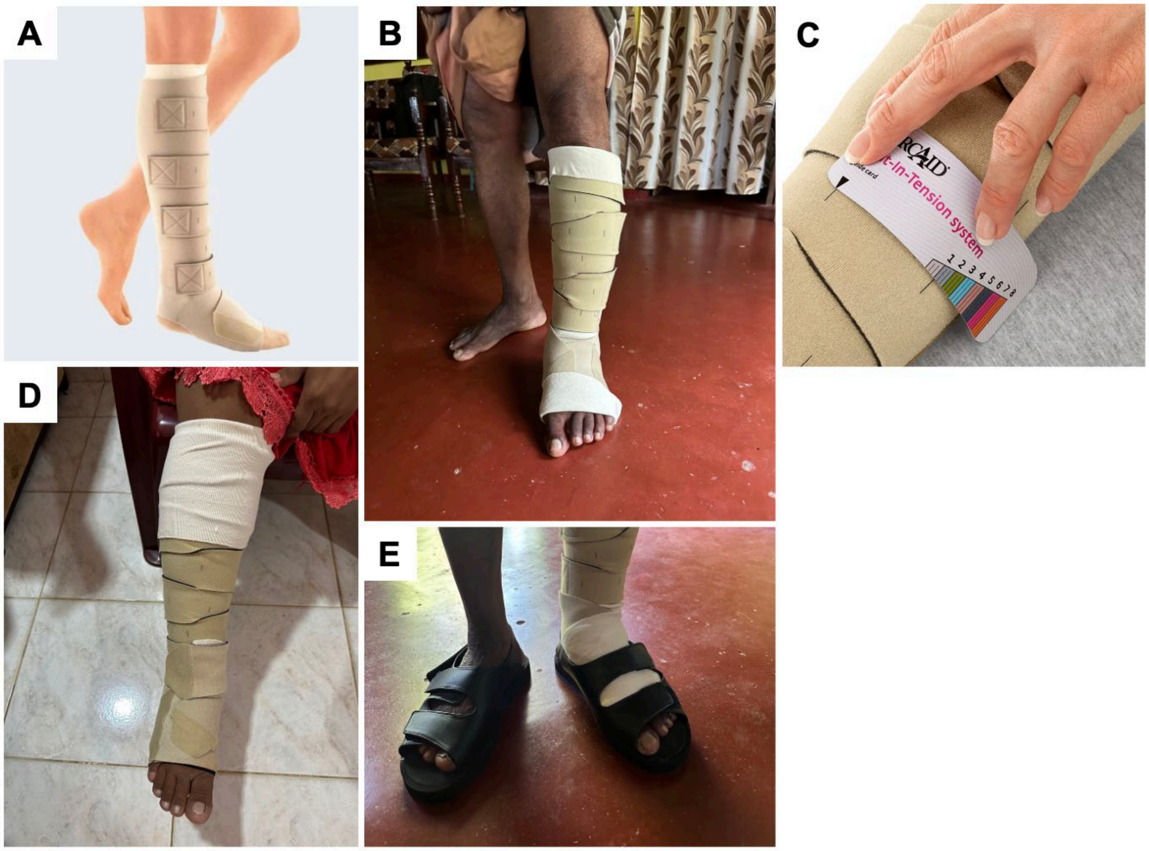

A.

Supplemental Figure A. Compression Garment Photos. Photos of the lower leg short stretch compression garment (Circaid ® Reduction Kit ™ SSCGs) and foot garment (Circaid ® Comfort PAC Band). (A) Photo from the medi USA catalog (https://mediusa-catalog.com/compression/circaid/juxtafit-leg/, accessed Dec 11, 2023). (B, D, E) Photos from study participants after donning the garment. Panel E also illustrates the adjustable footwear/shoes provided to protect the garment when walking outside of home. (C) Illustration of the tension markers and ruler used by participants to adjust the garment to provide uniform tension throughout the lower leg (taken from https://www.mediusa.com/healthcare-professional/lymphedema-solutions/healthcare-professional-products/circaid-reduction-kit/, accessed Dec 11, 2023).

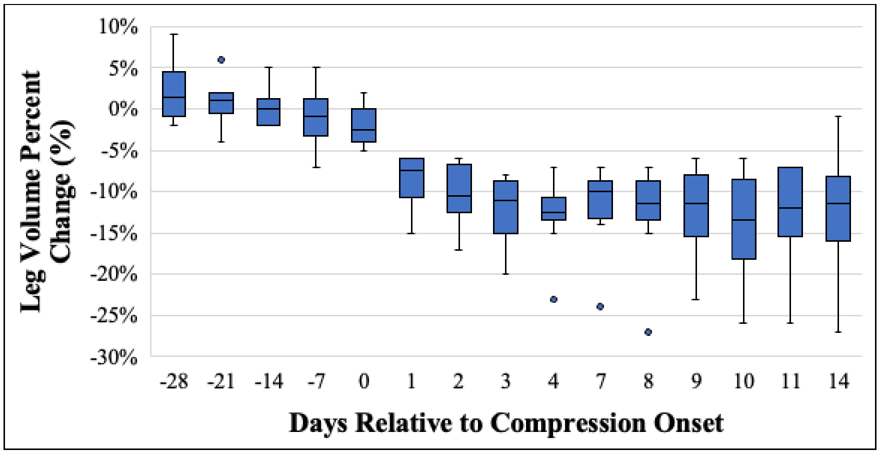

B.

Supplemental Figure B. Normalized Group Percent Volume Change. Group lower extremity median percent volume change was measured using a compilation of the within-subject percent changes at each visit and then calculated by normalizing each measurement in an individual to their pre-compression (day -28 to day 0) average lower extremity volume.

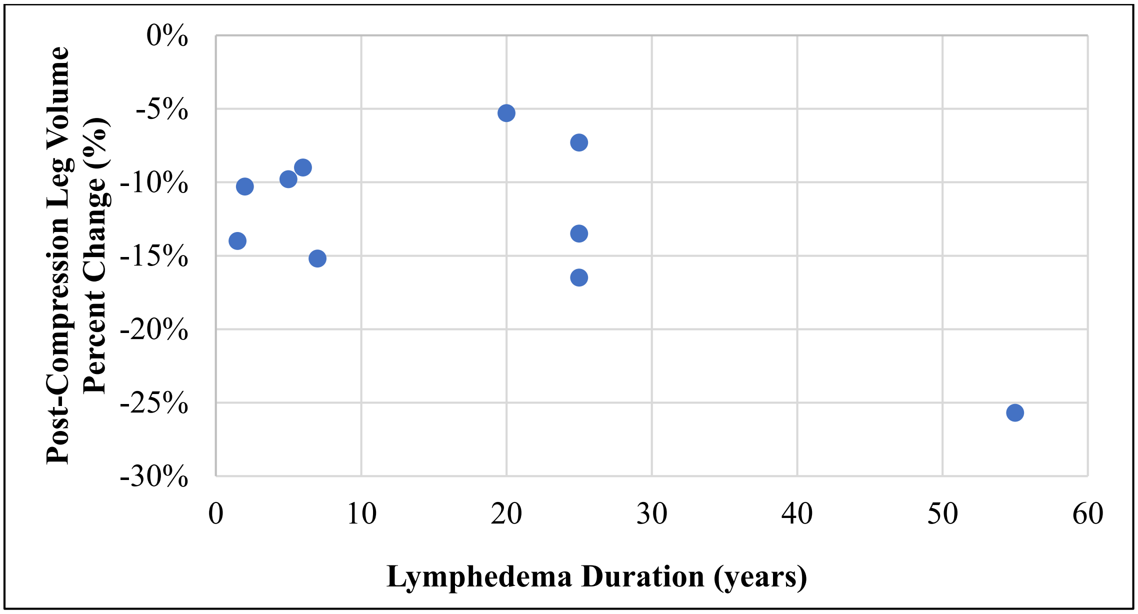

C.

Supplemental Figure C. Relationship Between Lymphedema Duration and Lower Extremity Volume Percent Change. Lymphedema duration of affected subject leg was self-reported by participants. Within-subject lower extremity volume percent change was calculated by normalizing each visit mean lower extremity volume to the pre-compression mean (day -28 to day 0) and post-compression leg volume percent change represents the post-compression average reduction. A negative leg volume percent change represents a reduction whereas a positive change represents a gain.
